# No association between sleep apnea, nocturnal blood pressure dipping, and cognitive performance among Swedish adults aged 50–65 years

**DOI:** 10.1111/joim.20104

**Published:** 2025-06-12

**Authors:** Xiao Tan, Mirjam Ljunggren, Johan Sundström, Eva Lindberg

**Affiliations:** ^1^ Department of Medical Sciences Respiratory, Allergy and Sleep Research Uppsala University Uppsala Sweden; ^2^ Department of Big Data in Health Science School of Public Health Department of Psychiatry Sir Run Run Shaw Hospital Zhejiang University School of Medicine Hangzhou China; ^3^ Department of Medical Sciences Uppsala University Uppsala Sweden; ^4^ The George Institute for Global Health University of New South Wales Sydney New South Wales Australia

**Keywords:** ambulatory blood pressure monitoring, cognitive performance, nocturnal blood pressure, sleep apnea, TMT‐B

Dear Editor,

Repeated breathing disruptions in obstructive sleep apnea (OSA) result in reduced oxygen supply to the brain and sleep deprivation. Previous studies have indicated that OSA is associated with impairments in several cognitive domains, including attention, executive function, and information processing speed [1, 2]. This may be explained by the neurological damage caused by intermittent hypoxia, as executive function and reaction time are thought to be related to the severity of hypoxemia [2]. However, a clinical trial has reported that continuous positive airway pressure treatment resulted in only mild and transient improvement in executive and frontal‐lobe function in severe OSA, but not in mild or moderate OSA [3]. This may be attributed to factors related to study design and participant selection, leading to a relatively low susceptibility to the neurocognitive effects of OSA and a reduced response to treatment, but it may also suggest the involvement of other underlying mechanisms. Normally, blood pressure (BP) decreases at nighttime, a phenomenon known as nocturnal BP dipping. However, when BP fails to decrease adequately or even increases, this is termed reduced or reverse dipping [4]. Results from a longitudinal study revealed that less nocturnal BP dipping was associated with worse cognitive function, especially executive function [5]. Reduced and reversed nocturnal BP dipping are particularly common among individuals with OSA [6], highlighting a potentially mediating effect of BP dipping in the association between sleep apnea and cognitive impairment.

Therefore, we aimed to determine the relationship between OSA and cognitive impairment and explore the potential mediation of this relationship by reverse BP dipping. We analyzed data from the population‐based Swedish CArdioPulmonary bioImage Study (SCAPIS), a random sample of adults aged 50–64 years. The details of this cohort study are provided in the Supporting Information. In this study, we included 2973 participants with complete data on overnight sleep breathing measurements, 24‐h ambulatory BP monitoring (ABPM), the Trail Making Test Part B (TMT‐B), and relevant covariates. OSA was quantified by the Apnea–Hypopnea Index (AHI). Cognitive function was assessed by TMT‐B, a timed executive function task requiring rapid alternation between connecting numbered and lettered circles, during the day after the overnight sleep breathing measurement [7]. Classification of AHI, ABPM parameters, category of dippers, and TMT‐B details are provided in the Supporting Information. To explore the dose–response association between severity of OSA or BP dipping and TMT‐B, restricted cubic spline models with three knots (at the 5th, 50th, and 95th percentiles) were performed, adjusting for age, sex, BMI, alcohol consumption, and smoking.

Characteristics of the study population by severity of OSA are presented in Table S1. Among all participants, 43% had OSA (AHI ≥ 5 events/h), 14% had moderate to severe OSA (AHI ≥ 15 events/h), and 4% had severe OSA (AHI > 30 events/h). After adjustment for potential confounders, we observed no independent association between OSA and cognitive performance assessed by TMT‐B (*p* = 0.599 for AHI, *p* = 0.335 for ODI) (Fig. 1a). There were no associations between sleep/wake systolic dipping ratio, sleep/wake diastolic dipping ratio, and cognitive performance (*p* = 0.077 for diastolic dipping ratio, *p* = 0.410 for systolic dipping ratio, Fig. 1b).

**Fig. 1:**
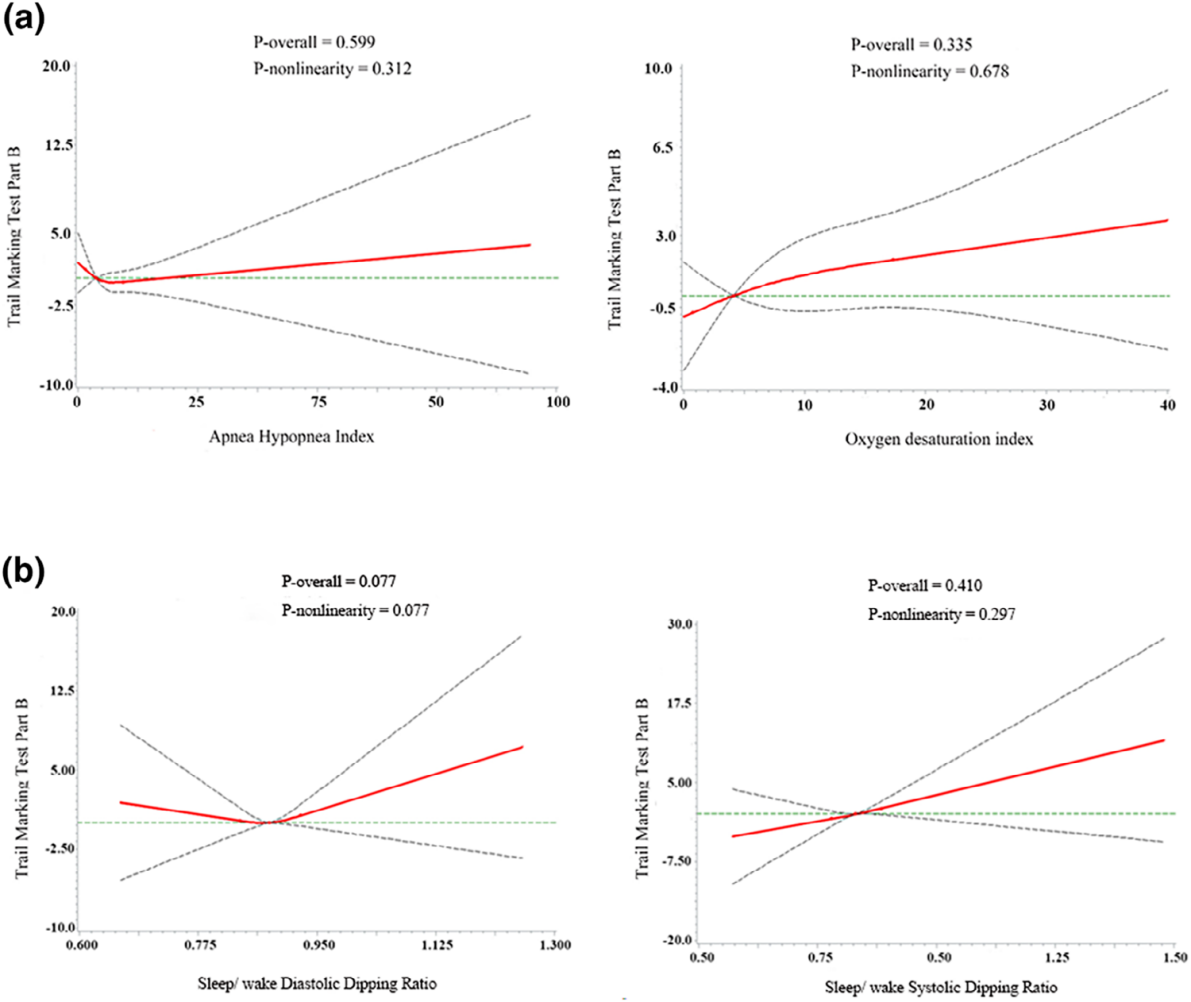
Association between (a) Apnea–Hypopnea Index, oxygen desaturation index; (b) diastolic dipping ratio, systolic dipping ratio, and Trail Making Test Part B in spline model. The spline model was adjusted for age, sex, BMI, alcohol consumption, and smoking status.

Our analysis revealed that neither AHI nor ODIs were associated with cognitive performance as assessed by TMT‐B when important confounders were considered. This is consistent with previous research [8], which also found no significant association between sleep‐disordered breathing and TMT‐B performance. However, our results contrast with findings from a recent study among US adults, which reported that mild‐to‐severe OSA was associated with poorer global cognition [9]. This may be a limitation of using the single cognitive test, which may not have been sensitive enough to detect potential associations between OSA and cognitive function. Additionally, participants in our study were relatively younger than those in a previous study linking OSA to cognitive impairment [9]. Moreover, unlike a previous longitudinal study using the Stroop test, a tool for assessing frontal lobe executive functions [5], we observed no association between nocturnal BP dipping patterns and cognitive performance in our population, which aligns with a study using the TMT‐B test among middle‐aged and older adults [10]. This discrepancy may result from differences in assessment focus, as both tests evaluate executive function with different emphases. It may also be indicative of a complex interplay between BP regulation and brain health, which cannot be fully captured by the dipping patterns alone. Additionally, the cross‐sectional design limits causal inference, and the nonsignificant trends between OSA, dipping patterns, and cognition may be due to limited power. Future studies with larger cohorts or longitudinal designs and comprehensive cognitive testing could explore the relationship between OSA and cognitive impairment and the potential mediating role of reverse BP dipping.

## Conflict of interest statement

The authors have no conflicts of interest related to this work.

## Supporting information




**Table S1**. Characteristics of the participants
**Figure S1**. Association between (A) Apnea Hypopnea Index, Oxygen desaturation index; (B) diastolic dipping ratio, systolic dipping ratio and Trail Making Test Part B in spline model. The spline model was adjusted for age, sex, BMI, alcohol consumption, smoking status, blood pressure medication and OSA treatment (continuous positive airway pressure treatment).

## Data Availability

The data that support the findings of this study are available on request from the corresponding author. The data are not publicly available due to privacy or ethical restrictions.
