# Supplementary material for: No association between sleep apnea, nocturnal blood pressure dipping, and cognitive performance among Swedish adults aged 50–65 years
Source: J Intern Med. 2025 Jun 12;298(2):135–7. doi: 10.1111/joim.20104 (PMC12239051; doi:10.1111/joim.20104)
Supplement: Supplementary file 1 — Table S1. Characteristics of the participants Figure S1. Association between (A) Apnea Hypopnea Index, Oxygen desaturation index; (B) diastolic dipping ratio, systolic dipping ratio and Trail Making Test Part B in spline model. The spline model was adjusted for age, sex, BMI, alcohol consumption, smoking status, blood pressure medication and OSA treatment (continuous positive airway pressure treatment). [file JOIM-298-135-s001.docx]

**Supplement method**

We analyzed data from the population-based Swedish CArdioPulmonary bioImage Study (SCAPIS), a collaborative research project between six Swedish universities including 30,154 adults aged 50–64 years [1]. Of the participants, 5,036 were investigated at the test centre in the city of Uppsala where the protocol was extended to also include a 24-hour Ambulatory blood pressure monitoring (ABPM). The participants in Uppsala were further invited to undergo a full night polygraphy sleep recording at home and a Trail Making Test Part B (TMT-B) that were performed by 4,038 and 3,877 participants, respectively. In this study, we included 2,973 participants with complete data on overnight sleep breathing measurements, ABPM, TMT-B and relevant covariates. The SCAPIS data collection was approved as a multi-centre study by the ethical review board in Umeå (# 2010-228-31M) and the present add-on study was approved by the ethical review board in Uppsala (# 2020-02113 0623). All participants provided written informed consent.

OSA was quantified by the Apnea-Hypopnea Index (AHI), categorized into three groups: AHI <5 events/hour, AHI 5-14.9 events/hour, and AHI >15 events/hour, and the oxygen desaturation index (ODI) (using desaturations 4%) assessed with ApneaLink (ResMed Corp., San Diego, CA, USA).

Ambulatory blood pressure was recorded using Labtech EC-3H/ABP monitors (Labtech Ltd, Debrecen, Hungary), set to measure every 30 minutes during the day (05:00–22:00) and at extended intervals (90 minutes) at night (22:00–05:00) to minimize sleep disruption. Time of ABPM readings were synchronized with sleep breathing measurement to distinguish blood pressure readings between sleep and wake states. Participants with a sleep-wake systolic or diastolic BP ratio of ≤0.90 were identified as normal/extreme systolic or diastolic dippers. Conversely, individuals with a sleep/wake BP ratio >0.90 but ≤1, and a ratio >1 were classified as reduced dippers and reverse dippers, respectively.

Cognitive function was assessed by Trail Making Test Part B (TMT-B) during the day after the overnight sleep breathing measurement. TMT-B involves connecting a series of circles that alternate between numbers and letters in alphabetical and numerical order (e.g., 1-A-2-B-3-C, etc.). Prior to commencing the TMT-B, participants were explained how to perform the task, including brief trial runs, to guarantee their comprehension of the procedure. Participants were instructed to complete these subtests as quickly as possible. The total time needed (in seconds) to correctly connect all symbols, which included the time to correct erroneously chosen paths was recoded. A shorter time taken to completing the test indicates better cognitive performance.

**References:**

1. Bergström G, Persson M, Adiels M, et al. Prevalence of Subclinical Coronary Artery Atherosclerosis in the General Population. Circulation. 2021;144(12):916-929. doi:10.1161/CIRCULATIONAHA.121.055340

**Supplement Table 1**. Characteristics of the participants

|  |  | **Apnea-hypopnea index, events/hr** | | | | ***P*-value^&^** |
| --- | --- | --- | --- | --- | --- | --- |
| **Characteristics** | **All** | **<5** | **5-14.9** | **≥15** |  | |
| **Number of participants** | 2973 | 1689 | 862 | 422 |  | |
| **Age, yrs** | 57.7±4.4 | 57.0±4.4 | 58.4±4.2 | 58.8±4.2 | <0.001 | |
| **Body mass index, kg/m^2^** | 26.8±4.2 | 25.6±3.6 | 27.9±4.2 | 29.7±4.9 | <0.001 | |
| **Sex, n (%)** |  |  |  |  | <0.001 | |
| **Women** | 1564 (52.6) | 1004 (59.4) | 422 (49.0) | 138 (32.7) |  | |
| **Men** | 1409 (47.4) | 685 (40.6) | 440 (51.0) | 284 (67.3) |  | |
| **Education, n (%)** |  |  |  |  | <0.001 | |
| **Elementary** | 200 (6.7) | 79 (4.7) | 75 (8.7) | 46 (10.9) |  | |
| **Secondary** | 1215 (40.9) | 644 (38.1) | 365 (42.3) | 206 (48.8) |  | |
| **College/University** | 1558 (52.4) | 966 (57.2) | 422 (49.0) | 170 (40.3) |  | |
| **Hypertension, n (%)** |  |  |  |  | <0.001 | |
| **No** | 2324 (78.2) | 1417 (83.9) | 631 (73.2) | 276 (65.4) |  | |
| **Yes** | 649 (21.8) | 272 (16.1) | 231 (26.8) | 146 (34.6) |  | |
| **Diabetes status, n (%)** |  |  |  |  | <0.001 | |
| **Normal glycemia** | 2202 (74.1) | 1338 (79.2) | 598 (69.4) | 266 (63.0) |  | |
| **Prediabetes** | 550 (18.5) | 261 (15.5) | 183 (21.2) | 106 (25.1) |  | |
| **Diabetes** | 221 (7.4) | 90 (5.3) | 81 (9.4) | 50 (11.9) |  | |
| **Leisure time physical activity, n (%)** |  |  |  |  | <0.001 | |
| **Sedentary** | 286 (9.6) | 126 (7.5) | 96 (11.1) | 64 (15.2) |  | |
| **Light** | 1361 (45.8) | 721 (42.7) | 433 (50.2) | 207 (49.1) |  | |
| **Moderate** | 969 (32.6) | 601 (35.6) | 252 (29.2) | 116 (27.5) |  | |
| **Intense** | 357 (12.0) | 241 (14.3) | 81 (9.4) | 35 (8.3) |  | |
| **Smoking status, n (%)** |  |  |  |  | 0.003 | |
| **Never** | 1759 (59.2) | 1051 (62.2) | 477 (55.3) | 231 (54.7) |  | |
| **Former** | 978 (32.9) | 509 (30.1) | 312 (36.2) | 157 (37.2) |  | |
| **Current** | 236 (7.9) | 129 (7.6) | 73 (8.5) | 34 (8.1) |  | |
| **Alcohol consumption, g/wk** | 7.1±6.2 | 6.5±5.7 | 7.6±6.8 | 8.3±6.8 | <0.001 | |
| **Sleep duration, min** | 379±114 | 385±109 | 371±118 | 374±123 | 0.006 | |
| **ODI, events/hr** | 7.2±8.8 | 2.6±2.2 | 8.7±4.3 | 22.9±12.4 | <0.001 | |
| **Mean systolic BP at awake, mmHg** | 128.2±11.4 | 126.3±10.9 | 129.9±11.6 | 132.1±11.3 | <0.001 | |
| **Mean systolic BP at sleep, mmHg** | 113.1±12.9 | 111.0±12.1 | 114.9±13.4 | 117.8±13.0 | <0.001 | |
| **Mean diastolic BP at awake, mmHg** | 80.7±7.8 | 79.5±7.6 | 81.7±7.9 | 83.2±7.9 | <0.001 | |
| **Mean diastolic BP at sleep, mmHg** | 67.1±8.8 | 65.8±8.4 | 68.1±8.9 | 70.3±8.9 | <0.001 | |
| **Systolic BP dipping pattern, n (%)*** |  |  |  |  | <0.001 | |
| **Normal and extreme dipper** | 1851 (62.3) | 1089 (64.5) | 533 (61.8) | 229 (54.3) |  | |
| **Non-dipper** | 949 (31.9) | 519 (30.7) | 268 (31.1) | 162 (38.4) |  | |
| **Reverse dipper** | 173 (5.8) | 81 (4.8) | 61 (7.1) | 31 (7.4) |  | |
| **Diastolic BP dipping pattern, n (%)*** |  |  |  |  | 0.011 | |
| **Normal and extreme dipper** | 2399 (80.7) | 1398 (82.8) | 681 (79.0) | 320 (75.8) |  | |
| **Non-dipper** | 498 (16.8) | 254 (15.0) | 157 (18.2) | 87 (20.6) |  | |
| **Reverse dipper** | 76 (2.6) | 37 (2.2) | 24 (2.8) | 15 (3.6) |  | |
| **Trail making test B result, s** | 88.2±32.3 | 86.2±32.1 | 89.3±31.2 | 94.1±34.1 | <0.001 | |

Data presented as mean±standard deviation or n (% in column).

ODI, oxygen desaturation index

*****Participants with a sleep-wake systolic or diastolic BP ratio of ≤0.90 were identified as normal/extreme systolic or diastolic dippers. Conversely, individuals with a sleep/wake BP ratio >0.90 but ≤1, and a ratio >1 were classified as non-dippers and reverse dippers, respectively.

**^&^**Anova was used for continuous variables and Pearson's Chi-squared test was used for categorical variables.

**
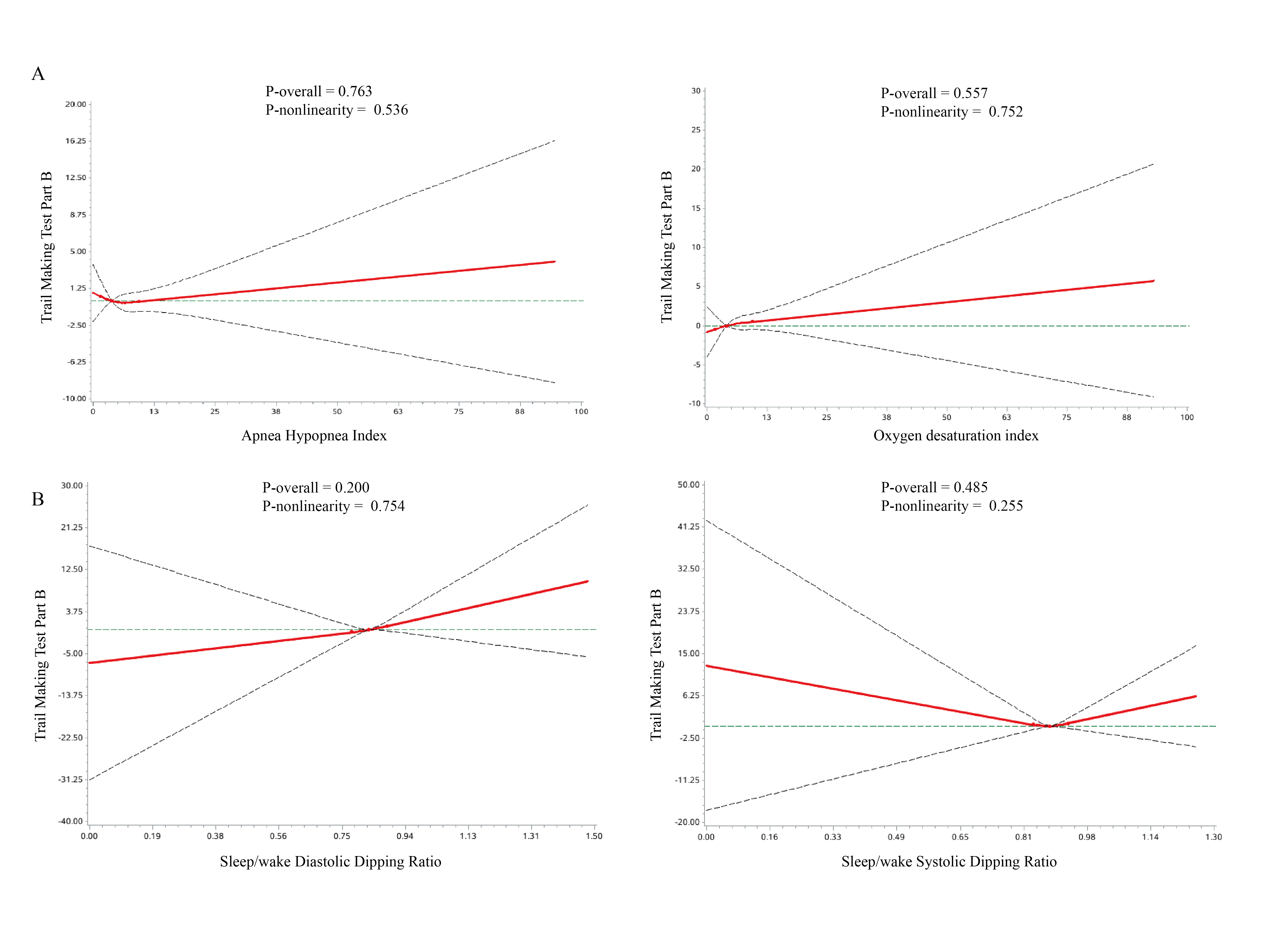
**

**Supplement Figure 1.** Association between (A) Apnea Hypopnea Index, Oxygen desaturation index; (B) diastolic dipping ratio, systolic dipping ratio and Trail Making Test Part B in spline model. The spline model was adjusted for age, sex, BMI, alcohol consumption, smoking status, blood pressure medication and OSA treatment (continuous positive airway pressure treatment).
